# Strain-specific immune response patterns to *Borrelia burgdorferi* infection: a comparative transcriptomic analysis in C3H and C57BL/6 mice

**DOI:** 10.1128/iai.00218-25

**Published:** 2026-02-17

**Authors:** Guozhong Zhou, Yan Dong, Huangjuan Zhao, Yu Zhang, Yantong Chen, Meng Liu, Yanshuang Luo, Aihua Liu, Fukai Bao

**Affiliations:** 1 Department of Pathogen Biology and Immunology, Faculty of Basic Medical Sciences, Kunming Medical University 71240, Kunming, Yunnan, China; 2 Department of Pain Medicine, The Affiliated Anning First People's Hospital of Kunming University of Science and Technology 629636 , Kunming, Yunnan, China; 3 School of Basic Medical Sciences, Kunming University of Science and Technology 47910, Kunming, Yunnan, China; 4 The Institute for Tropical Medicine, Kunming Medical University 71240, Kunming, Yunnan, China; Tulane University, New Orleans, Louisiana, USA

**Keywords:** *Borrelia burgdorferi*, Lyme arthritis, immune cell infiltration, transcriptome, differentially expressed genes

## Abstract

*Borrelia burgdorferi* (*Bb*), transmitted through tick vectors, induces Lyme arthritis (LA), with disease progression intimately correlated with host genetic characteristics. Laboratory investigations have demonstrated marked disparities in infection responses among distinct mouse strains: C57BL/6 mice have mild arthritis and rapid tissue repair, whereas C3H mice exhibit severe arthritic manifestations. Comparing these strains has helped identify genetic and immune factors important for arthritis development. In this study, female C57BL/6 and C3H mice were inoculated with *Bb* via bilateral footpad injection. Disease progression was evaluated through multidimensional parameters, including joint swelling measurements, radiographic examinations, and histopathological analyses at acute (14 days) and chronic (56 days) phases. RNA-seq of joint tissue, combined with single-sample gene set enrichment analysis, immune deconvolution, and multi-omics enrichment revealed strain-divergent signatures. The experimental data unveiled strain-specific immune response patterns: C3H mice exhibited persistent inflammatory responses characterized by heightened complement system activation, sustained inflammatory mediator expression, and prolonged inflammasome activity. C57BL/6 mice maintained relatively stable inflammatory mediator levels and immune homeostasis. Transcriptomic analysis revealed 2,183 (C3H) and 439 (C57BL/6) differentially expressed genes on day 14 post-infection, encompassing processes related to immune cell recruitment, cytokine networks, and complement activation. These findings illuminate the regulatory role of host genetic background in temporal characteristics of immune responses, providing novel molecular insights into differential susceptibility to LA.

## INTRODUCTION

Lyme disease, caused by *Borrelia burgdorferi* (*Bb*) infection, represents a complex pathological entity whose causative agent necessitates alternating transmission between vertebrate hosts (predominantly rodents and avian species) and tick vectors to sustain its life cycle. Upon human infection through tick bites, the pathogen can induce late-stage Lyme arthritis (LA), presenting a significant clinical challenge in endemic regions (1). Patients typically manifest recurrent or persistent inflammation of major joints, particularly the knee, accompanied by edema, algesia, and restricted mobility. In the absence of appropriate therapeutic intervention, certain cases may progress to refractory chronic inflammation (2–6).

The pathogenic mechanisms of LA remain incompletely understood. Beyond direct bacterial infection, persistent inflammation involves a complex interplay of residual antigens, latent infections, and host genetic susceptibility. Even after the eradication of live bacteria, lingering peptidoglycan fragments continue to provoke the immune system. Certain pathogens may enter a dormant state or reside in immunologically privileged sites, evading detection while their antigens persistently activate immune responses (7–9). Animal studies have demonstrated that aberrant amplification of type I interferon signaling in joint tissues is closely associated with severe inflammatory phenotypes (10, 11). Genetic determinants exert pivotal regulatory influences on disease progression. Specific variations in the HLA system correlate with antibiotic treatment efficacy, underscoring the influence of genetic background on therapeutic outcomes (12, 13). This intricate regulatory network elucidates the observed clinical heterogeneity, patients manifest varying degrees of disease severity and treatment responses, shaped by the unique interplay of genetic predisposition, immune status, and pathogen characteristics.

Previous investigations have predominantly relied on *in vitro* experimental paradigms, which insufficiently elucidated the complex and nuanced interaction networks inherent within living biological systems. To surmount this limitation, we devised a research protocol utilizing two mouse strains. Two experimental animal lineages with distinct susceptibilities to *Borrelia burgdorferi* were selected: C57BL/6 (mild symptoms) and C3H (severe symptoms). This experimental design emerged from preliminary observations demonstrating that mice with diverse genetic backgrounds exhibit distinctive immunological response characteristics following infection (14–19). The investigation employed a longitudinal tracking strategy, conducting transcriptomic analyses at two crucial temporal points: the acute inflammatory phase (14 days post-infection) and the recovery phase (56 days post-infection), aiming to identify and characterize the micro-environmental features and molecular mechanisms influencing disease progression. This comparative methodological approach not only promises to elucidate host-pathogen interaction molecular patterns but also provides theoretical foundations for developing targeted therapeutic strategies, ultimately enhancing treatment outcomes for LA patients.

## MATERIALS AND METHODS

### Experimental animals

Female mice aged 4–6 weeks, weighing 22–24 g, were utilized across two distinct strains: C3H/HeNCr (C3H) and C57BL/6NCr (C57BL/6). The experimental subjects were procured from Beijing Vital River Laboratory Animal Technology Co., Ltd. and maintained in specific-pathogen-free facilities at the Basic Medical College of Kunming Medical University. Housing conditions were meticulously regulated: ambient temperature was maintained at 21.5°C, with alternating 12-h light-dark cycles. Animals were provided *ad libitum* access to water and food, with bedding materials renewed daily. A total of 48 mice were employed in this investigation, with 24 allocated to each strain. Within each strain cohort, 12 subjects were sacrificed specifically for histopathological assessment through histological staining, while the remaining 12 were sacrificed for RNA extraction and subsequent sequencing.

### Pathogen culture and infection protocol

The low-passage *Bb* strain B31 (DSMZ, Germany) underwent a meticulous tripartite cultivation process. The B31 strain was chosen due to its common usage in laboratory settings and prior experience (20, 21). Initially, bacterial revitalization was achieved by homogenizing 0.5 mL bacterial suspension with 9.5 mL Barbour-Stoenner-Kelley II medium (22), followed by incubation at 37°C under a 5% CO_₂_ atmosphere for 72 h. Upon reaching optimal bacterial density (60–120 HPF), the culture underwent amplification in 50 mL fresh medium for an additional 7-day period. The final preparation phase involved suspending cultured organisms in phosphate-buffered saline (PBS), followed by enumeration, viability assessment, and precise concentration adjustment. For model establishment, mice received bilateral footpad injections (50 μL per footpad) containing *Borrelia burgdorferi* spirochetes (1 × 10^6^ spirochetes) suspended in PBS. The high inoculum of 1 × 10^6^ was used to ensure consistent infection across all mice. The control cohorts were administered equivalent volumes of PBS.

### Disease assessment protocol

Comprehensive phenotypic analyses were conducted at two pivotal time points, day 14 (acute phase) and day 56 (recovery phase) post-infection, employing three distinct methodologies. Ankle joint swelling was quantified by precisely measuring the anteroposterior diameter of the rear ankle joints using digital calipers, thereby assessing the degree of joint edema from days 14 to 56. On day 14 (acute phase), microscopic evaluation of joint tissue specimens stained with H&E was performed for detailed assessment of articular damage and inflammatory infiltration patterns. Furthermore, arthritis severity was blindly scored based on inflammatory cell infiltration, synovial cell hyperplasia, and fibroplasia. Inflammatory cell infiltration was graded as 0 (none or minimal), 1 (<15 cells/HPF), 2 (15–30 cells/HPF), or 3 (>30 cells/HPF). Synovial cell hyperplasia was scored as 0 (none), 1 (monolayer swelling), 2 (bilayer swelling), or 3 (multilayer swelling). Fibroplasia was graded as 0 (none), 1 (mild), 2 (moderate), or 3 (severe). This scoring system allowed for a semi-quantitative assessment of arthritis severity based on key histological features. High-resolution radiographic imaging of the rear ankle joints was also performed on day 14 (acute phase) to assess osteoarticular pathological manifestations and structural modifications, providing a thorough evaluation of disease progression and overall severity. All experiments were independently repeated in triplicate.

### Molecular and bioinformatic analysis protocol

#### RNA extraction and sequencing

Following established methodology (11, 23), each mouse’s posterior tibiotarsal joints were isolated by removing the skin from the lower leg and cutting 3–5 mm above and below the joint; approximately 80 mg of the tissue was excised. During transection, primary osseous structures were diligently circumvented to mitigate the risk of bone marrow contamination. Subsequently, the tibiotarsal joint, together with its adjacent articular capsule and associated connective tissues, was meticulously isolated. Both ankle joints of each mouse were combined, homogenized in ice-cold PBS, and immediately stored at −80°C. Total RNA extraction was performed utilizing MolPure Cell/Tissue Total RNA Kit (#19221ES50, Shanghai Yeasen Biotechnology).

#### Bioinformatic analysis pipeline

Molecular characterization of joint tissues was conducted at distinct temporal points (days 14 and 56 post-infection) with three mice per group. Paired-end sequencing (PE150) was performed on the Illumina NovaSeq 6000 platform by Hangzhou Lianchuan Biotechnology. Quality assessment was performed via FastQC, followed by read trimming and adapter removal using Trimmomatic. Processed reads were aligned to the mouse reference genome (mm10) using HISAT2, while transcript assembly and quantification were accomplished through StringTie. Differential expression analysis employed DESeq2, with significance thresholds set at |log_2_FoldChange| > 1 and adjusted *P* < 0.05 (day 14) or *P* < 0.05 (day 56).

#### Functional annotation and enrichment analysis

Initial functional enrichment analysis of differentially expressed genes was conducted using the DESeq2 package on raw data. Differential gene selection criteria were established as |log_2_FoldChange| > 1 with adjusted *P*-value < 0.05 on day 14 post-infection, and |log_2_FoldChange| > 1 with *P*-value < 0.05 on day 56 post-infection. For the identified differentially expressed genes, two complementary approaches were employed for functional enrichment analysis: first, utilizing the online analytical platform Metascape (https://metascape.org/gp/index.html#/main/step1) for Gene Ontology (GO) functional annotation analysis (24); second, implementing R packages “clusterProfiler” and “org.Mm.eg.db” for GO functional annotation and KEGG pathway enrichment analysis (25). GO analysis encompassed three dimensions: biological processes (BPs), cellular components (CCs), and molecular functions (MFs), with a significance threshold set at *P*-value < 0.05 (26).

Gene Set Enrichment Analysis (GSEA) was executed using the gseKEGG function from the clusterProfiler package. Differentially expressed genes were ranked by log₂(FC) values, converted to ENTREZ IDs, and subjected to enrichment analysis within the mouse KEGG database (nPerm = 10,000, gene set size 10–200, *P* < 0.05). Enrichment results were visualized using the gseaplot2 function, highlighting pathways with the most significant enrichment scores.

#### Immune cell infiltration analysis

We used single-sample gene set enrichment analysis (ssGSEA) aiming to estimate immune cell infiltration across various data sets. The scores were calculated using the expression levels of immune cell-specific marker genes (27, 28) and delineated immune infiltration levels in each sample, quantifying the density of 28 immune cell types, including Activated B cells, activated CD4 T cells, activated CD8 T cells, activated dendritic cells, CD56bright natural killer cells, CD56dim natural killer cells, central memory CD4 T cells, central memory CD8 T cells, effector memory CD4 T cells, effector memory CD8 T cells, eosinophils, gamma delta T cells, immature B cells, immature dendritic cells, myeloid-derived suppressor cells (MDSCs), macrophages, mast cells, memory B cells, monocytes, natural killer T cells, natural killer cells, neutrophils, plasmacytoid dendritic cells, regulatory T cells, T follicular helper cells, type 1T helper cells, type 17T helper cells, and type 2T helper cells.

#### Immunohistochemistry

Formalin-fixed, paraffin-embedded tissue blocks were sectioned at 4 μm thickness. Selected sections underwent H&E staining for histopathological assessment. For immunohistochemical analysis, paraffin sections were deparaffinized and rehydrated. They underwent antigen retrieval in citrate buffer for 10 min, followed by PBS rinsing. After blocking with 3% BSA (30 min, room temperature), sections were incubated with primary antibodies overnight at 4°C: CD11b (1:300 dilution, catalog GB15058, Servicebio). Following PBS washes, sections received secondary antibody incubation for 1 h at room temperature. Visualization used DAB chromogen for 10 min, with subsequent hematoxylin counterstaining. Sections were then dehydrated and permanently mounted. Cellular positivity was determined by pale yellow to brown cytoplasmic or nuclear granules. All stained slides were scanned on AxioScan Z1 (Zeiss, Germany) software, analyzed by Aipathwell digital pathology image analysis software (Servicebio) for positive cell density (number/mm²) scoring.

### Statistical analysis

Statistical analyses were performed using R 3.6.3. Inter-group comparisons employed Student’s *t*-test or Wilcoxon rank-sum test. Differential expression significance was established at fold change > 2.0 and *P* < 0.05, with *P*-value adjustment via false discovery rate (FDR) correction.

## RESULTS

### Assessment of infection status and arthritis severity

#### Radiographic findings

**Fig 1:**
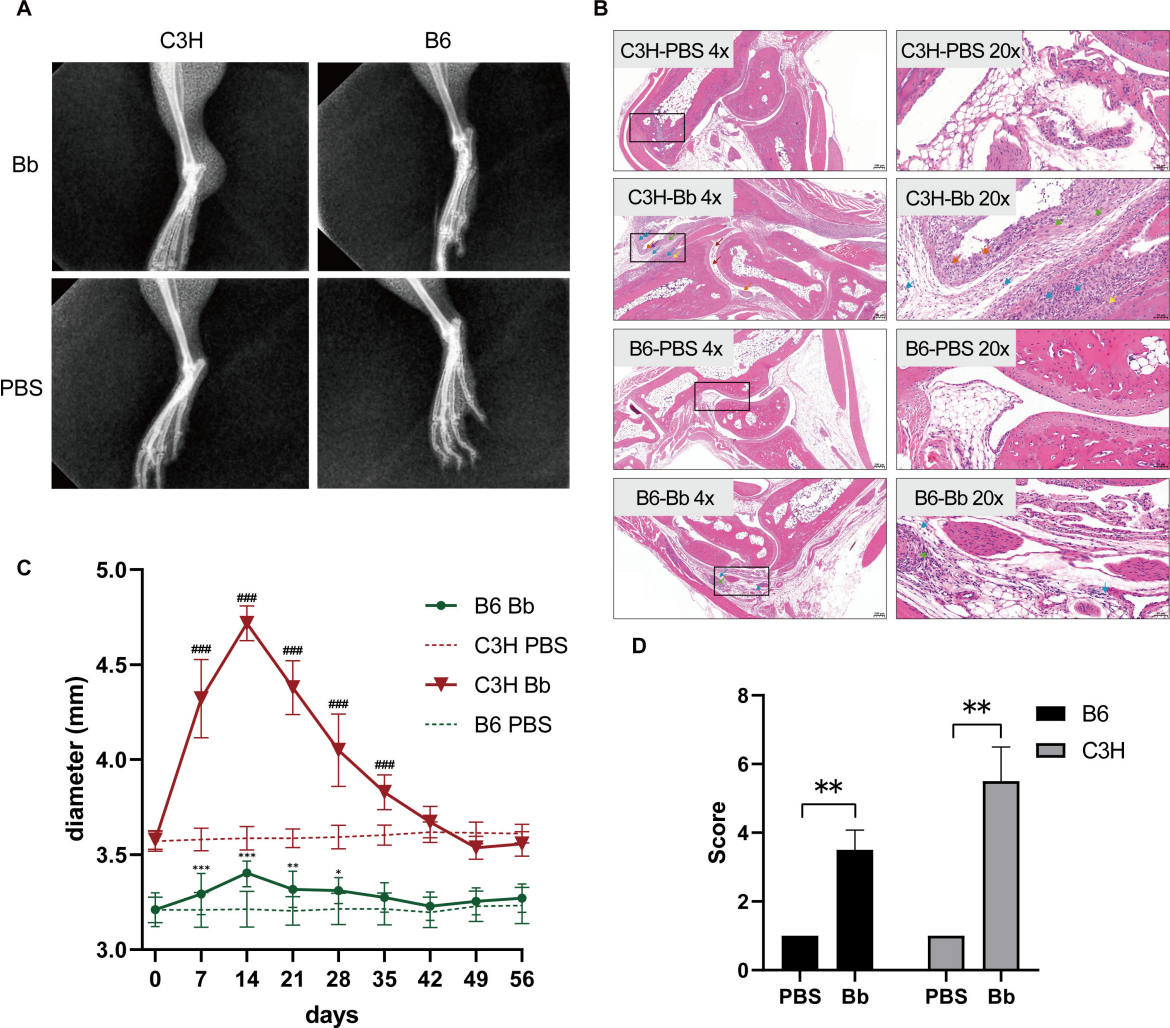
Characteristic features of lyme arthritis in different mouse strains after *Borrelia burgdorferi* infection. (**A**) X-ray examination of the hind limb joints on day 14 after infection to assess soft tissue edema in the joints. The upper row is the infection group, and the lower row is the PBS control group. (**B**) H&E-stained joint tissue on day 14 post-infection demonstrates inflammatory cell infiltration and tissue edema. Orange arrows mark pronounced synovial cell hyperplasia with structural disorganization. Green arrows indicate extensive connective tissue proliferation replacing native architecture. Purple arrows designate scattered necrotic cellular debris. Yellow arrows identify focal bone erosions. Blue arrows show dense inflammatory cell infiltration throughout joint tissue. Red arrows highlight isolated inflammatory cells within joint space. The left panels are at 4× magnification, while the right panels are magnified at 20×, showcasing detailed enlargements of specific regions within the 4× images. Scale bars: 200 μm at 4× magnification, and 50 μm at 20× magnification. (**C**) Dynamic changes in the diameter of the rear ankle joints of mice (C3H and C57BL/6) within 56 days after infection. The diameter of the joint of the hind limb was measured weekly using a digital caliper, and the results are expressed as mean ± standard error (*n* = 6/group, *P < 0.05 and **P < 0.01 compared to C57BL/6 control group, ^#^*P* < 0.05 and ^##^*P* < 0.01 compared to C3H control group). (**D**) Pathological severity scores in H&E-stained joint tissue from C3H and C57BL/6 mice (*P < 0.05 and **P < 0.01). Regarding the rectangular areas detected by the forensic tools, this is attributable to our utilization of whole slide imaging in pathology, which constructs digital imaging by stitching together square fields of view and supports free magnification from 0× to 400×. Blank areas within the scanning range (where there is no tissue on the slide) are identified as a white background.

Radiographic examination (Fig. 1A) on day 14 post-infection revealed significant soft tissue edema in C3H mice, while C57BL/6 mice showed no appreciable edematous changes.

#### Histopathological evaluation

H&E staining (Fig. 1B, day 14) revealed synovial/soft tissue inflammation in infected versus PBS-control strains. Both control strains exhibited intact synovial architecture: surface synovial cells overlay loose connective tissue without hyperplasia or inflammation; articular cavities remained pristine with smooth cartilage and healthy chondrocytes. C3H-infected mice demonstrated severe pathology: synovial hyperplasia (orange arrows), connective tissue hyperplasia (green arrows), necrotic debris (purple arrows), osseous erosions (yellow arrows), extensive inflammation (blue arrows) with eosinophilic material, and red arrows highlight isolated inflammatory cells within the joint space. Conversely, C57BL/6-infected mice showed only mild connective hyperplasia (green arrows) and focal lymphocytic infiltration (blue arrows) with preserved joint integrity. Pathological severity scores are shown in Fig. 1D.

#### Joint diameter measurements

As illustrated in Fig. 1C, following *Bb* infection, the rear ankle joint inflammation manifested in C3H and C57BL/6 mice from day 2 post-infection, reaching peak intensity around week 2, with gradual resolution occurring over 4–5 weeks. C3H mice exhibited the most pronounced articular edema, followed by C57BL/6 mice, which demonstrated only minimal swelling.

### Transcriptomic analysis overview

**Fig 2:**
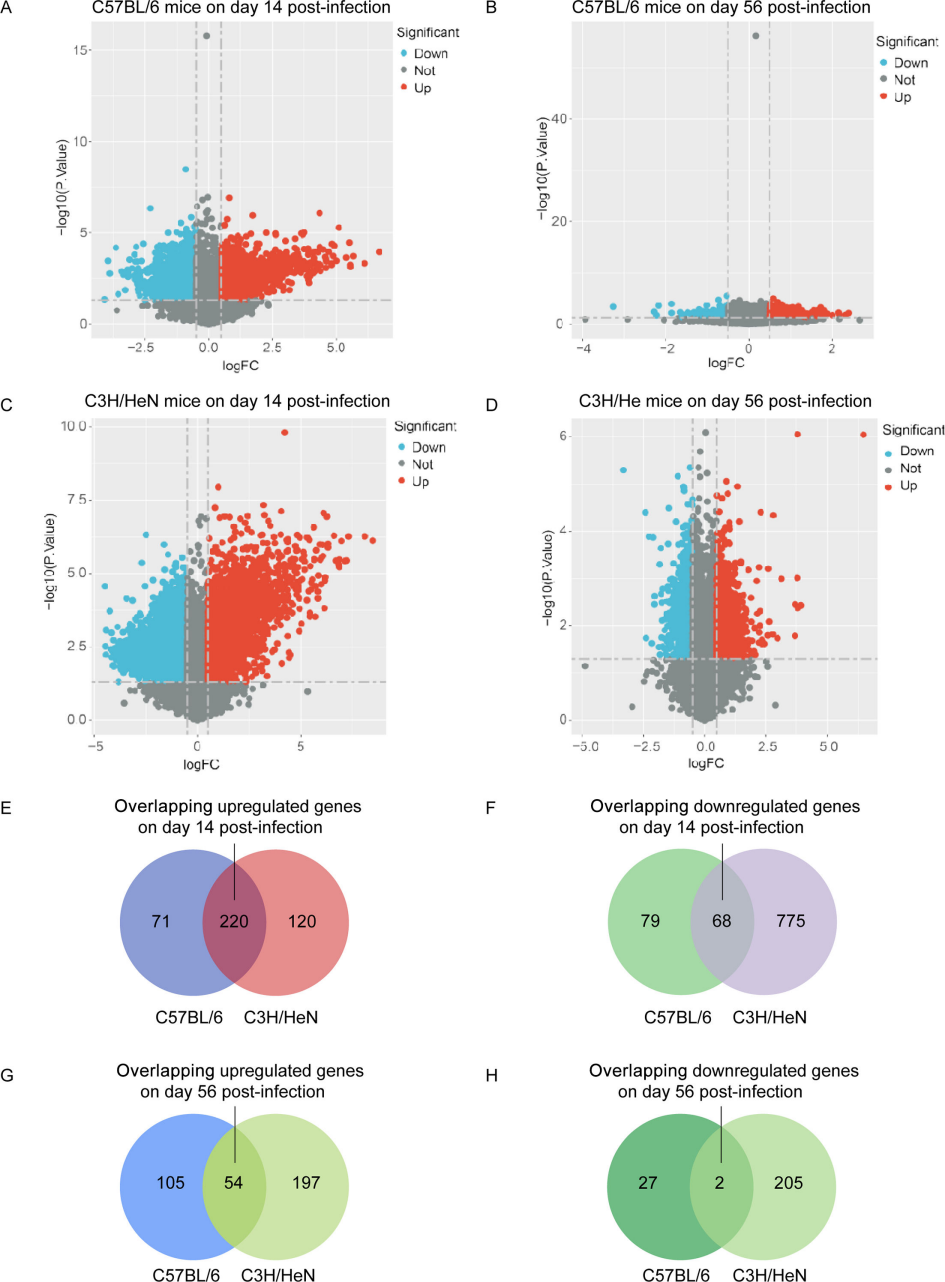
Transcriptomic differential analysis of different mouse strains after *Borrelia burgdorferi* infection. (**A–D**) RNA sequencing analysis of gene expression profiles in the joint tissues of C57BL/6 (B6) and C3H/HeN (C3H) mice on days 14 and 56 after infection. The data for each mouse strain are presented in the form of a volcano plot, where the red and blue dots represent significantly upregulated and downregulated genes, respectively. (**E–H**) Venn diagrams showing the overlap of differentially expressed genes among the two mouse strains at different time points. Panels E and F show the distribution of differentially expressed genes on day 14 after infection, with panel E representing upregulated genes and panel F representing downregulated genes; panels G and H show the distribution of differentially expressed genes on day 56 after infection, with panel G representing upregulated genes and panel H representing downregulated genes. The numbers indicate the number of differentially expressed genes in each region.

This investigation aimed to elucidate differential gene expression patterns among distinct mouse strains during infection, with particular emphasis on C3H and C57BL/6 mice in the context of LA. Transcriptional alterations in articular tissues were comprehensively documented on days 14 and 56 post-infection, as visualized through volcano plots (Fig. 2A through D). On day 14 post-infection, utilizing stringent selection criteria of absolute log fold change (abs[log_2_FC]) > 1 and adjusted *P*-value < 0.05, the following differential expression patterns emerged (Table 1): C3H mice exhibited 2,183 significantly modulated transcripts, while C57BL/6 mice demonstrated 439 differentially expressed genes. This pattern indicates a more robust transcriptional response in C3H mice during the acute phase of infection. By day 56 post-infection, employing criteria of abs (log₂[FC]) > 1 and *P*-value < 0.05, C3H mice exhibited 458 significantly modulated transcripts, suggesting persistent immune activation, whereas C57BL/6 mice showed more modest changes with 188 differentially expressed genes. This observation potentially reflects sustained inflammatory activity in C3H mice during the chronic phase of infection.

**TABLE 1:** Number of unique gene transcripts changed >2-fold in the joints of C3H and B6 mice at 14 and 56 days post-infection

	Arthritis phenotype	14 days			56 days		
C57BL/6 (B6)	Mild	439 (all)	291 (up)	148 (down)	188 (all)	159 (up)	29 (down)
C3H/HeN (C3H)	Severe	2,183 (all)	1,340 (up)	843 (down)	458 (all)	251 (up)	207 (down)

### Venn diagram analysis

Analysis of differentially expressed genes on day 14 post-infection revealed substantial overlap among C3H and C57BL/6 mouse strains: 220 genes were commonly upregulated across both strains, while 68 genes were consistently downregulated (as illustrated in Fig. 2E and F). In contrast, by day 56 post-infection, the transcriptional convergence was markedly reduced, with only 54 genes commonly upregulated across strains and 2 significant overlaps observed among downregulated genes (as depicted in Fig. 2G and H).

### GO enrichment analysis

#### GO enrichment analysis of common differentially expressed genes

**Fig 3:**
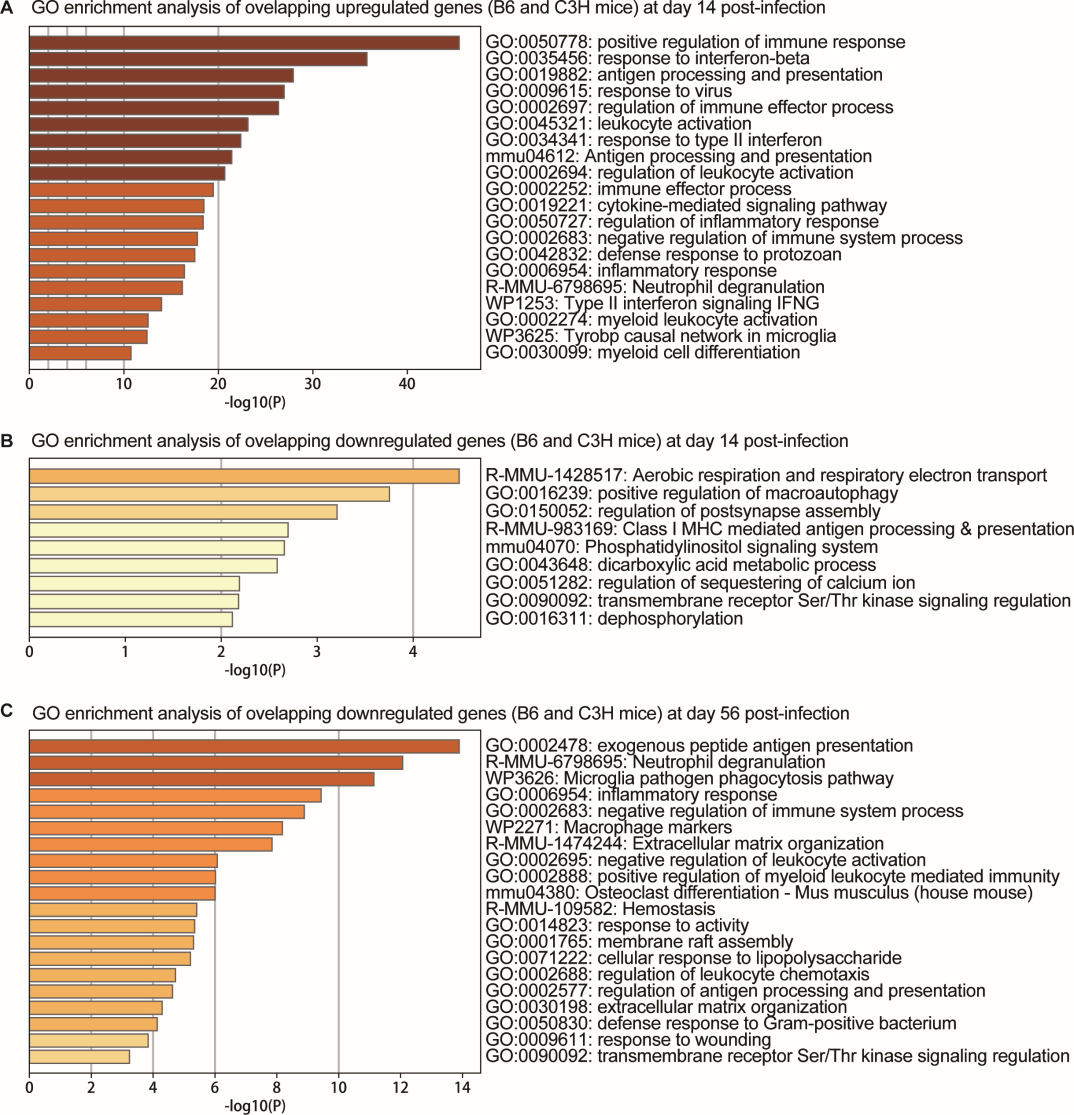
GO functional enrichment analysis of differentially expressed genes common across different mouse strains after infection. The GO enrichment for commonly upregulated genes 14 days after infection (**A**); GO enrichment for commonly downregulated genes 14 days after infection (**B**); GO enrichment for commonly upregulated genes 56 days after infection (**C**); the length of the bars represents the degree of enrichment, and the color intensity indicates the significance level (-log_10_[*P*-value]).

GO enrichment analysis of commonly upregulated genes on day 14 post-infection highlighted enrichment in multiple immune response-related pathways, encompassing both innate and adaptive immunity. Key cellular processes included leukocyte and myeloid cell activation (GO:0045321 and GO:0002274) and neutrophil degranulation (R-MMU-6798695). At the molecular level, pathways involved cytokine production and signaling (GO:0019221), antigen processing and presentation (GO:0019882), and interferon responses (WP1253, GO:0035456). Notably, the activation of negative immune system regulators (GO:0002683) suggested sophisticated immune balance mechanisms. The commonly downregulated genes primarily involved positive regulation of macroautophagy (GO:0016239) and Class I MHC-mediated antigen processing and presentation (R-MMU-983169) (as illustrated in Fig. 3A and B).

By day 56 post-infection, enriched pathways encompassed immune defense mechanisms, including antigen processing (GO:0002478 and GO:0002577), innate effector functions (neutrophil degranulation R-MMU-6798695, microglial phagocytosis WP3626), inflammatory response (GO:0006954), bacterial-specific defense (GO:0050830), leukocyte regulation (chemotaxis GO:0002688, activation control GO:0002695, and myeloid immunity GO:0002888), and immune modulation (GO:0002683) (as illustrated in Fig. 3C).

#### GO enrichment analysis of mouse strain-specific responses

**Fig 4:**
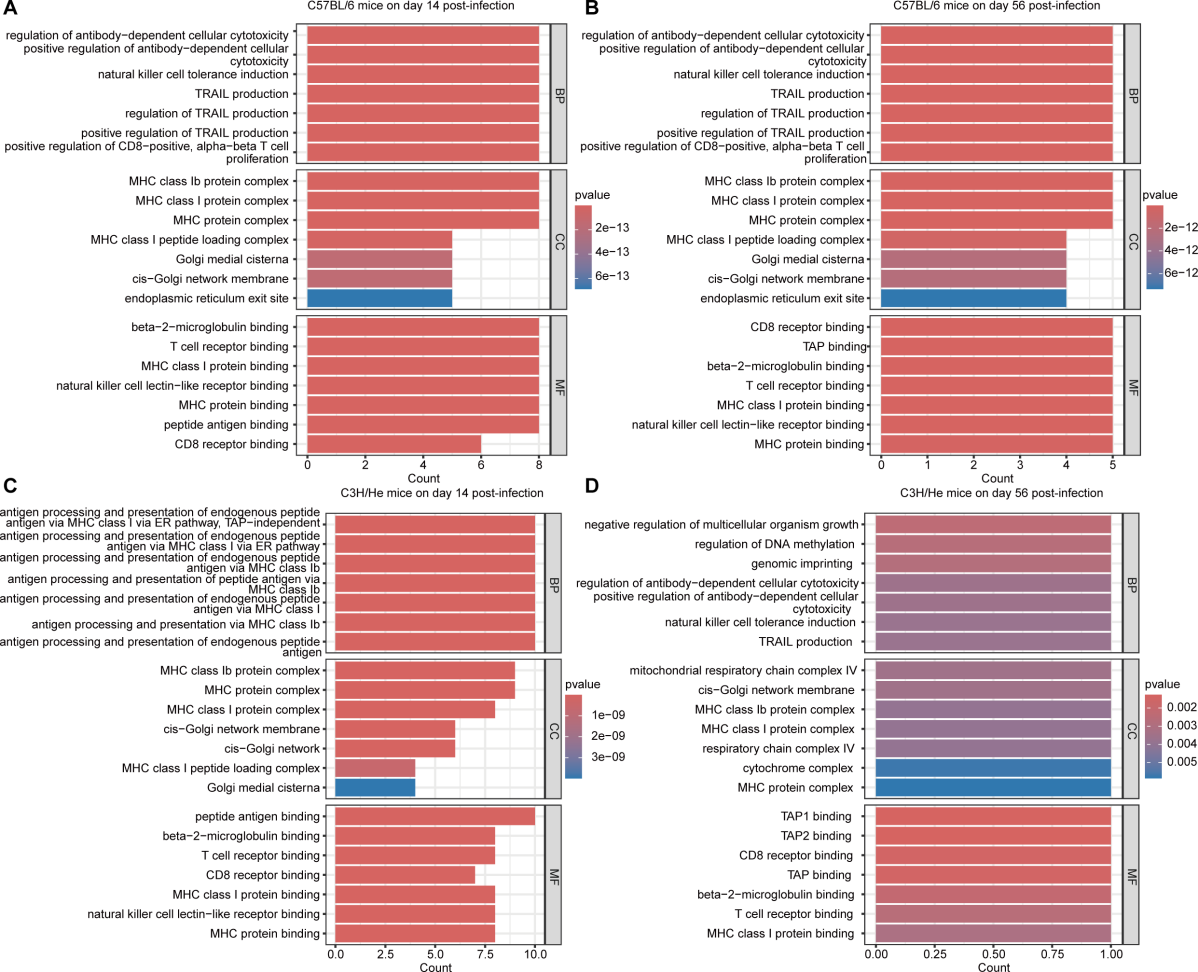
GO functional enrichment analysis of differentially expressed genes in different mouse strains after *Bb* infection. (**A–D**) The GO functional enrichment results for C57BL/6 (**A and B**) and C3H/HeN (**C and D**) mice on days 14 and 56 after infection are shown. Each subplot displays the significantly enriched functional terms (*P* < 0.05) at the levels of BP, CC, and MF. The length of the bars represents the degree of enrichment, and the color intensity indicates the significance level (*P*-value). The *x*-axis shows the gene count, and the *y*-axis displays the enriched functional terms.

Comparative GO enrichment analysis across C57BL/6 and C3H mice infected with *Bb* revealed distinct yet overlapping immune response patterns on days 14 and 56 post-infection (as illustrated in Fig. 4A through D). All strains demonstrated sustained enrichment in key cellular components, particularly the Golgi network and MHC protein complexes, indicating active antigen processing and presentation machinery. At the molecular function level, common enriched pathways included beta-2-microglobulin binding, T cell receptor binding, natural killer cell receptor binding, and CD8 receptor binding.

Strain-specific variations were notable: C57BL/6 mice showed unique enrichment in TRAIL production and antibody-dependent cell cytotoxicity regulation, with dynamic changes in peptide antigen-binding pathways between early and late phases. C3H mice displayed the most comprehensive immune response, characterized by pronounced endogenous antigen processing via MHC I and Ib pathways on day 14, evolving into a more complex regulatory profile by day 56, including active type IIa hypersensitivity pathways.

### KEGG pathway enrichment analysis

**Fig 5:**
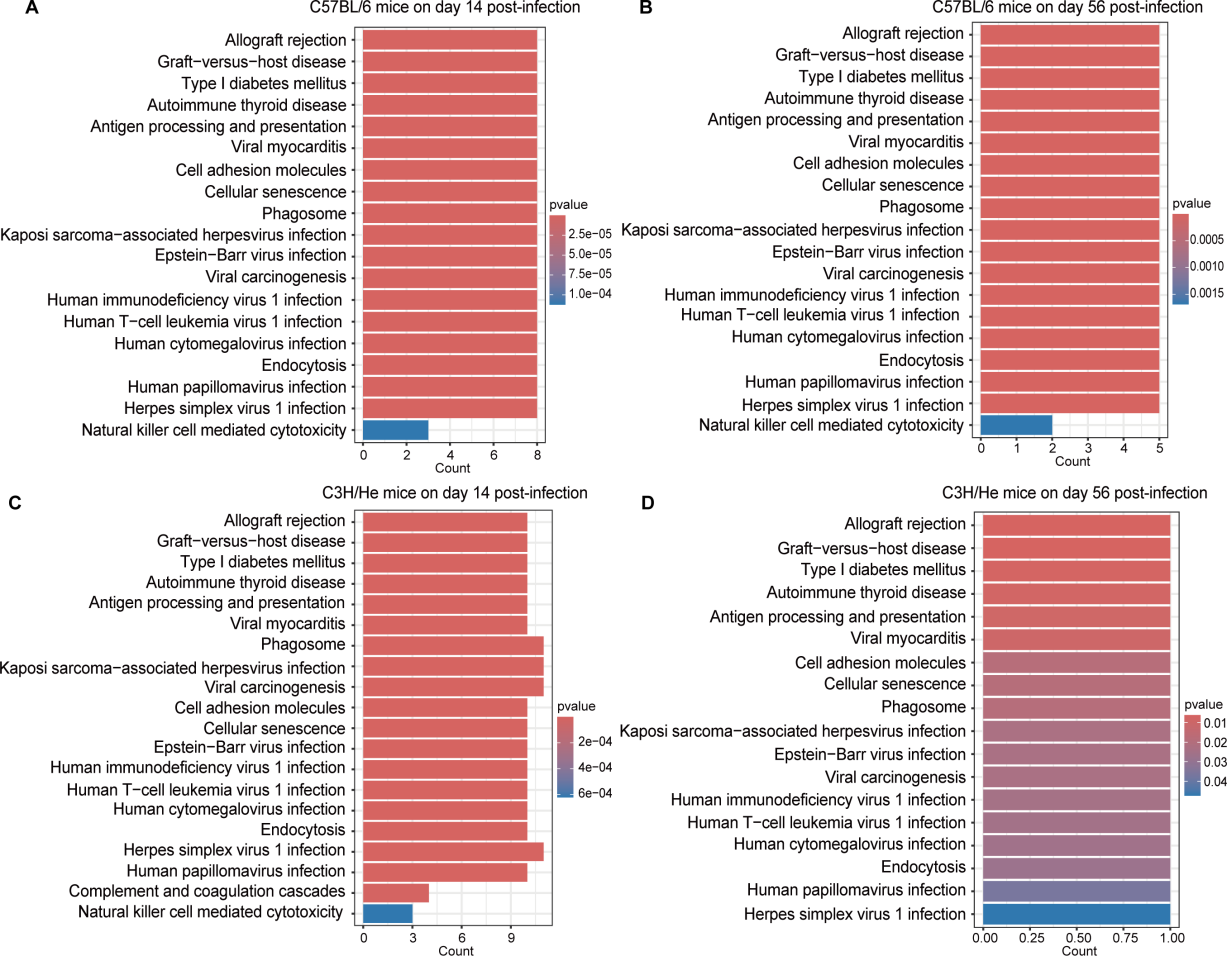
KEGG pathway enrichment analysis of differentially expressed genes in different mouse strains after *Bb* infection. (**A–D**) The KEGG pathway enrichment results for C57BL/6 (**A and B**) and C3H/HeN (**C and D**) mice on days 14 and 56 after infection are shown. The bar plots display the significantly enriched signaling pathways (*P* < 0.05), where the bar length represents the enrichment factor (Rich Factor), and the color intensity indicates the significance level (*P*-value). The *x*-axis shows the number of differentially expressed genes in the enriched pathways, and the *y*-axis lists the names of the significantly enriched signaling pathways. All pathways are analyzed based on the latest annotations in the KEGG database.

Based on KEGG pathway enrichment analysis of C57BL/6 and C3H mouse strains infected with *Bb*, we observed significant enrichment in pathways, including viral myocarditis, antigen processing and presentation, autoimmune thyroid disease, type I diabetes, graft-versus-host disease, and allograft rejection across two strains. This suggests that *Bb* infection triggers common immune response mechanisms that transcend mouse strain differences (Fig. 5).

### GSEA enrichment analysis

**Fig 6:**
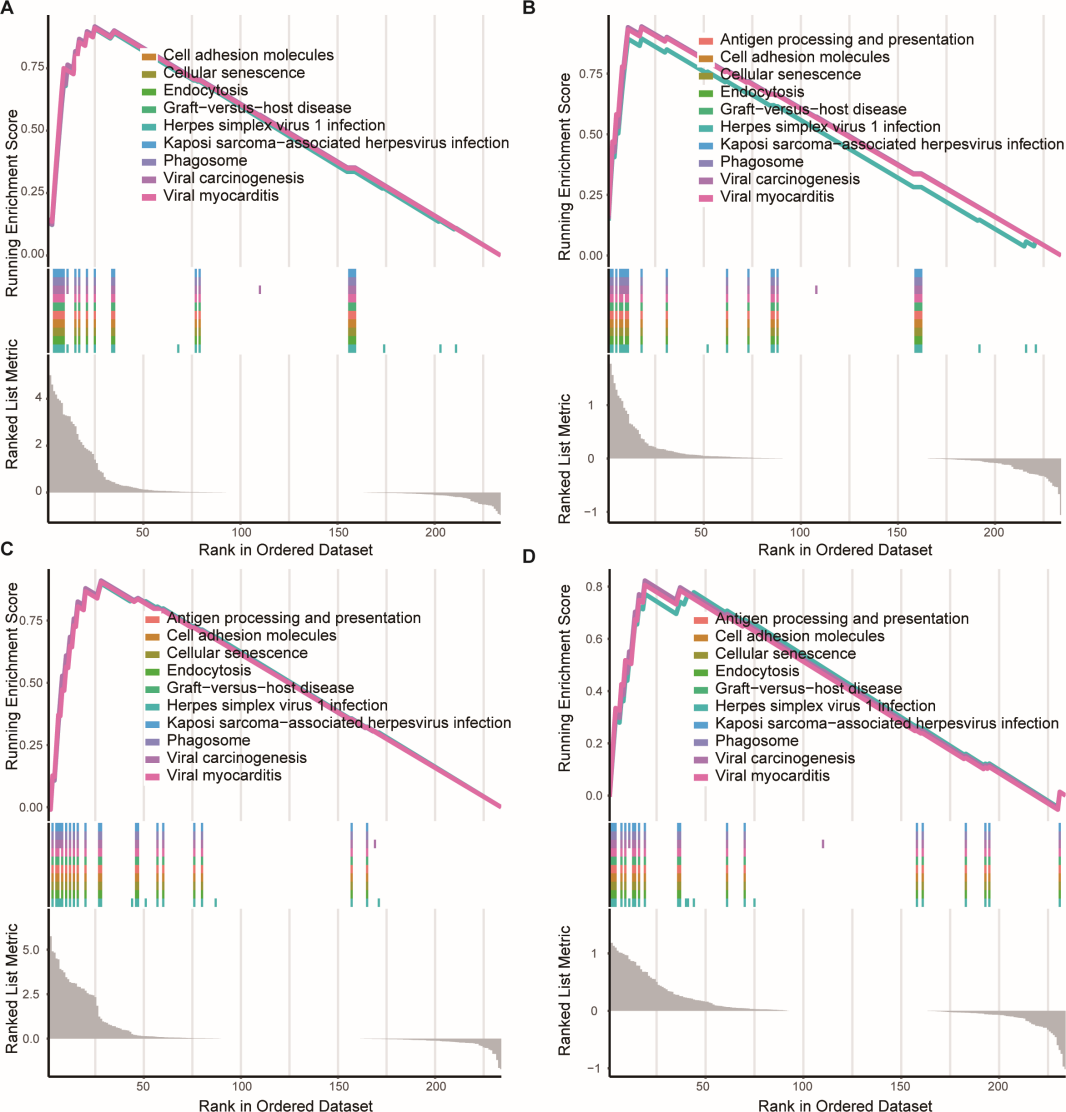
GSEA enrichment analysis results of different mouse strains after *Bb* infection. Panels **A**–**D** show the GSEA enrichment plots for C57BL/6 (**A and B**) and C3H/HeN (**C and D**) mice on days 14 and 56 after infection, respectively. Each subplot contains three parts: the colored curve at the top shows the cumulative change trend of the enrichment score of different pathways along the gene sorting position; the colored vertical lines in the middle represent the distribution of the genes in the gene set among all the sorted genes; the gray bar graph at the bottom shows the sorting distribution of all genes according to the degree of differential expression (Ranked List Metric). All significantly enriched gene sets meet the default GSEA criteria (*P* < 0.001, FDR < 0.25). *The top 10 most significant functional pathways are displayed for each time point.

GSEA enrichment analysis revealed remarkably similar immune responses to *Bb* infection across two mouse strains (C3H and C57BL/6), demonstrating comparable complex dynamic changes in immune responses following infection. At both early (day 14) and late (day 56) stages of infection, both mouse strains exhibited significant regulation of specific pathways: upregulated pathways primarily involved inflammatory responses, immune responses, and virus-related processes, such as graft-versus-host disease, phagosome function, and viral myocarditis. Conversely, downregulated pathways focused on immune regulatory mechanisms, including antigen processing, cell adhesion, and cellular senescence. This sustained pathway modulation reflects the dynamic adjustment of the host immune system in response to *Bb* infection, highlighting the complexity and adaptability of immune responses at different stages of infection (Fig. 6).

### Analysis of gene expression changes in different mouse strains following *Bb* infection

This study compared immune responses in C57BL/6 and C3H mice at 14 and 56 days post-*Bb* infection (Table 2). C3H mice exhibited early, robust immune activation at 14 days, including strong complement induction (C2: log_₂_FC = 3.56, C4b: log_₂_FC = 3.93, Cfp: log_₂_FC = 3.26, and C3: log_₂_FC = 4.31) and cytokine/chemokine upregulation (Csf2: log_₂_FC = 13.85, Ccl8: log_₂_FC = 5.72, Cxcl16: log_₂_FC = 5.07, Il12a: log_₂_FC = 2.03, and Lta: log_₂_FC = 3.69). They also showed coordinated pro-inflammatory (Tnf: log_₂_FC = 7.07) and anti-inflammatory (Il10: log_₂_FC = 7.35 and Tgfb1: log_₂_FC = 1.76) signaling, with Nlrp3 upregulation (log_₂_FC = 4.43) linked to joint swelling. Adaptive immunity was robust in C3H, marked by T-cell (Cd3d: log_₂_FC = 6.78 and Cd3g: log_₂_FC = 7.18), B-cell (Cd79a: log_₂_FC = 4.06), and checkpoint (Ctla4: log_₂_FC = 4.99) activation.

**TABLE 2:** Expression levels of selected gene set changed in the joints of C57BL/6 and C3H mice at 14 and 56 days post-infection^*a*^

	14 days				56 days			
	C57BL/6		C3H		C57BL/6		C3H	
	log_2_FC	*P*-value	log_2_FC	*P*-value	log_2_FC	*P*-value	log_2_FC	*P*-value
Chondrocyte development								
Acan	−0.66	0.54	−1.2	0	0.86	0.19	−0.18	0.55
Axin2	−1.14	0.07	−1.44	0	0.34	0.17	−0.98	0.06
Chsy1	0.06	0.82	0.65	0.05	0.61	0	0.14	0.3
Col11a1	−5.31	0.42	6.45	0.33	1.95	0.3	0	1
Ext1	0.93	0.05	0.23	0.75	0.31	0.66	0.79	0.26
Matn1	−0.66	0.06	−0.2	0.07	0.21	0.06	−0.61	0.01
Pthlh	−0.41	0.38	−0.05	0.92	1.88	0.09	−0.1	0.83
Rflna	−0.19	0.57	0.36	0.28	0.34	0.04	0.02	0.89
Runx2	1.44	0.09	1.26	0.01	1.64	0.02	0.8	0.01
Sfrp2	2.85	0.22	3.99	0	1.51	0.05	1.81	0
Osteoclast proliferation								
Csf1	1.05	0.14	1.8	0	1.15	0.06	0.57	0.11
Nmbr	0.11	0.66	1.15	0	−0.21	0.4	0.11	0.71
Npr3	−3.06	0	−3.64	0.02	−0.12	0.66	−1.19	0.03
Ocstamp	0.46	0.41	1.31	0.02	0.86	0.27	0.94	0.33
Tcirg1	2.02	0.15	3.76	0	1.36	0.07	0.77	0.03
Tnfsf11	1.34	0.09	2.86	0.02	2.07	0.14	−0.35	0.65
Adherens junction assembly								
Actb	−0.51	0.05	0.12	0.3	0.01	0.96	0.08	0.44
Jam3	−0.55	0.02	0.1	0.46	−0.08	0.6	−0.25	0.04
Pak2	0.65	0.22	1.28	0	0.22	0.13	0.18	0.29
Vcl	−0.64	0.13	−1.29	0.04	−0.82	0.53	−0.57	0.08
Zfp703	0.05	0.82	1.02	0.01	0.39	0.31	0.17	0.2
Chemokine binding								
Ccr1	−1.13	0	−0.17	0.39	−0.19	0.43	−0.71	0
Ccr2	7.99	0.42	11.09	0.06	7.83	0.42	0	1
Ccr3	−0.2	0.33	0.69	0.01	−0.05	0.71	−0.1	0.4
Ccr4	−0.98	0.01	−0.59	0.03	−0.1	0.58	−0.84	0
Ccr5	9.93	0.42	5.01	0.01	0	1	12.6	0.19
Ccr6	−0.01	0.93	0.32	0.07	−0.12	0.65	0.09	0.51
Ccr7	−0.61	0.03	0.49	0.26	0.45	0.21	0.13	0.43
Ccr8	−0.77	0.18	−0.47	0.47	−0.24	0.71	−0.79	0.37
Ccr9	−0.26	0.33	−1.16	0.23	0.53	0.23	−0.33	0.1
Ccrl2	−0.32	0.23	0.1	0.55	0.22	0.23	−0.32	0.03
Cxcr2	−0.74	0	−0.5	0.01	0.09	0.33	−0.2	0.01
Cxcr4	−0.25	0.44	0.93	0.3	0.54	0.03	0.56	0.02
Chemokine receptors bind chemokines								
Ccl20	0.69	0.24	−0.71	0.28	1.34	0.31	0.71	0.24
Cd74	0.18	0.24	0.47	0.01	0	0.97	0.52	0.07
Clnk	−0.54	0.08	−0.5	0.07	0.41	0.09	0.09	0.62
Fcer1g	−1.23	0	−0.99	0.01	0.47	0.4	0.34	0.75
Foxp3	−1.11	0	−0.15	0.34	0.05	0.76	−0.47	0.03
Ifng	−0.37	0.51	−0.19	0.54	0.07	0.79	−0.09	0.79
Il10	13.51	0.29	7.35	0.04	−8.27	0.42	9.42	0.21
Il1b	0.42	0.38	0.5	0.15	0.88	0.07	0.46	0.08
Il21	−1.65	0.02	−1.82	0	−0.23	0.55	−0.5	0.03
Il6	−1.02	0	−0.72	0	−0.39	0.05	−0.65	0.01
Mif	−0.16	0.49	−0.17	0.82	0.03	0.82	−0.03	0.82
Myd88	0.89	0.12	−0.69	0.35	0.27	0.22	0.37	0.59
Nlrp3	3.42	0.11	4.43	0.02	0.23	0.5	0.75	0.13
Tgfb1	1.24	0.1	1.76	0	0.94	0.07	0.8	0.04
Tlr4	−0.56	0	−0.24	0.05	0.03	0.71	−0.45	0.02
Tnf	5.69	0.2	7.07	0.01	1.67	0.26	1.85	0.04
Trem1	0.25	0.63	−0.84	0.3	1.37	0.05	1.15	0.09
Xcl1	−6.56	0.42	−7.29	0.21	5.9	0.42	5.9	0.42
Activation of innate immune response								
Aim2	−1.03	0	−2.01	0	−0.09	0.16	−0.6	0.01
Bcl10	0.76	0.35	1.32	0	0.22	0.58	0.25	0.18
Cd274	5.6	0.15	7.86	0	−0.59	0.45	1.42	0.14
Cd86	1.42	0.17	3.53	0	1.24	0.1	0.78	0.01
Cgas	5.67	0.42	−5.48	0.42	0	1	0	1
Clec4n	2.45	0.16	5.33	0.01	0.94	0.24	1.61	0.01
Cyba	2.88	0.11	4.48	0	1.45	0.04	1.47	0.06
Eif2ak2	1.76	0.1	2.39	0	0.64	0.07	0.61	0.1
Fosl1	0.17	0.49	0.67	0.05	0.39	0.24	0.64	0.03
Gbp3	0.56	0.2	1.16	0.03	0.55	0.12	0.13	0.44
Gbp5	0.42	0.54	−0.2	0.37	1.07	0.22	−0.65	0.49
Ifih1	−0.85	0	−0.32	0.16	−0.17	0.12	0	0.97
Igtp	−0.49	0.5	2.3	0.07	2.06	0.26	0.39	0.45
Inava	0.47	0.36	−0.36	0.62	1.14	0.02	0.35	0.4
Irak2	−0.18	0.81	1.58	0.09	1.7	0.23	0.89	0.37
Irak3	0.96	0.01	1.57	0.08	0.37	0.18	0.89	0.02
Irf1	4.14	0.12	4.76	0.09	0.19	0.72	0.69	0.26
Irf2	−0.91	0	−1.5	0	−0.21	0.37	−0.02	0.88
Irf4	0.93	0.03	0.67	0.1	1.51	0.01	0.43	0.36
Irf7	0.89	0.59	−0.7	0.42	0.35	0.74	0.08	0.89
Irgm1	0.26	0.42	0.77	0.05	0.05	0.83	0.42	0.03
Irgm2	−1.57	0	−0.72	0.01	−0.1	0.51	0.13	0.5
Klri2	−0.18	0.39	−3.71	0	0.14	0.77	0.26	0.69
Klrk1	2.47	0.09	3.31	0.02	1.68	0.02	1.31	0.15
Mefv	5.39	0.17	6.65	0.02	2.23	0.03	1.78	0.15
Nlrp6	−0.3	0.56	0.4	0.44	0.56	0.49	0.44	0.61
Nod2	−0.38	0.59	−0.42	0.65	−0.32	0.51	0.3	0.67
Nr1d1	−1.06	0.06	−0.64	0.06	0.35	0.11	−0.27	0.06
Nr1h4	9.78	0.35	10.42	0.15	0	1	7.61	0.42
Oasl1	7.09	0.42	0	1	7.09	0.42	0	1
Rftn1	0.53	0.11	1.16	0.02	0.29	0.29	0.57	0.29
Ripk2	0.07	0.73	0.51	0.04	0.48	0.01	0.13	0.07
Rnf135	0.87	0.14	1.37	0	−0.08	0.73	0.58	0.08
Smpdl3b	−0.97	0.01	−0.08	0.48	−0.24	0.08	−0.42	0
Sting1	1.37	0.14	2.36	0	0.19	0.59	0.79	0.15
Tbk1	0.97	0.19	2.12	0	0.47	0.21	0.69	0.01
Ticam1	1.15	0.4	−0.94	0.34	−0.99	0.24	0.16	0.85
Tlr12	10.05	0.22	14.79	0	0	1	−0.35	0.57
Tlr2	2.09	0.09	2.1	0	1.25	0.01	1.14	0.01
Tlr3	−0.35	0.57	−1.81	0.19	−0.15	0.51	−1.03	0.05
Tlr5	−0.2	0.56	0.14	0.54	0.27	0.4	−0.08	0.65
Adaptive immune response								
Ada	2	0.08	2.79	0.01	0.67	0.26	1.21	0.04
Alcam	1.88	0.07	3.02	0	1.2	0.16	1	0.07
Arg2	4.51	0.21	6.99	0.01	−1.68	0.3	1.12	0.26
B2m	−9.05	0.35	0.23	0.88	0	1	−8.68	0.19
Batf	0.73	0.04	0.39	0.38	0.61	0.14	1.21	0
Cd19	−0.13	0.7	0.62	0	0.12	0.55	−0.1	0.76
Cd28	1.71	0.25	3.81	0.04	−0.1	0.8	0.37	0.21
Cd3d	−0.65	0	6.78	0	−0.51	0.53	1.34	0.14
Cd3e	1.3	0.4	−3.51	0.45	−0.61	0.78	−0.55	0.75
Cd3g	4.63	0.2	7.18	0.01	2.44	0.33	2.13	0.23
Cd4	5.52	0.06	5.74	0.02	3.13	0.18	0.02	0.96
Cd40	2.57	0.13	4.3	0	1.09	0.18	1.57	0.24
Cd79a	2.13	0.06	4.06	0	3.59	0.21	−1.08	0.11
Cd8a	0.36	0.82	−0.92	0.49	2	0.53	1.9	0.34
Ctla4	3.21	0.2	4.99	0.02	1.56	0.13	1.47	0.19
Ctsc	0.89	0.31	0.41	0.17	0.08	0.91	−0.15	0.72
Cx3cr1	1.15	0.34	1.78	0.01	0.74	0.19	−0.55	0.64
Dbnl	1.2	0.09	1.91	0	0.32	0.02	0.61	0
Gata3	0.57	0.44	1.98	0	−0.48	0.62	0.71	0.24
H2-Ab1	4.03	0.04	5	0.01	0.7	0.18	1.29	0.06
H2-D1	9.32	0.42	5.09	0.1	1.5	0.42	1.77	0.13
Complement activation								
C1qa	−0.29	0.22	−0.66	0.25	0.41	0.15	−0.67	0.02
C2	2.88	0.14	3.56	0	1.5	0.15	1.18	0.01
C3	3.24	0.1	4.31	0	1.25	0.17	1.03	0.12
C4b	6.25	0.12	3.93	0.05	2.11	0.04	1.29	0.21
Cd46	−0.69	0.23	−0.9	0.08	−0.06	0.92	0.22	0.73
Cd55b	0.36	0.28	−0.91	0.09	0.55	0	0.14	0.1
Cfb	−8.05	0.42	0.17	0.93	−8.29	0.42	0.22	0.91
Cfp	2	0.03	3.26	0	0.86	0.07	0.61	0.12
Ighg3	0	1	10.27	0.42	0	1	−10.04	0.42
Masp2	−0.34	0.44	0.19	0.48	0.96	0.01	−0.09	0.7
Cytokine production involved in immune response								
Card9	0.18	0.47	1.01	0	0.18	0.32	0.3	0.09
Casp1	1.82	0.03	1.63	0	0.72	0.12	1.13	0
Malt1	−7.75	0.42	2.66	0.37	−0.04	0.98	−6.28	0.42
Pycard	2.75	0.13	2.97	0.01	9.9	0.34	0.77	0.67
Slc11a1	−0.05	0.56	−0.04	0.73	0.09	0.15	−0.04	0.38
Tnfrsf1b	0.39	0.19	0.95	0.06	0.47	0.25	1.24	0.14
Tnfsf4	0.27	0.6	0.62	0.19	0.43	0.41	0.42	0.04
Cytokine receptor binding								
Ccl24	−1.92	0.05	−4.5	0.06	1.47	0.01	−0.58	0
Ccl5	2.69	0.04	3.66	0.01	−1.85	0.41	2.01	0.07
Ccl7	0.52	0.48	0.06	0.93	−0.47	0.38	0.42	0.55
Ccl8	4.75	0.05	5.72	0	1.15	0.02	1.66	0.09
Csf2	1.98	0.36	13.85	0	0	1	0.88	0.56
Csf3	−1.88	0.01	−3.06	0.05	1.04	0.18	−1.89	0.03
Cxcl1	3.47	0.36	9	0.01	7.33	0.42	1.03	0.49
Cxcl10	1.29	0.18	2.62	0	0.73	0.25	0.77	0
Cxcl11	−0.1	0.61	−0.42	0.22	0.68	0.01	0.25	0.12
Cxcl12	−0.67	0	−0.11	0.4	−0.17	0.25	−0.11	0.08
Cxcl16	3.48	0.09	5.07	0	1.31	0.14	1.94	0.09
Cxcl17	0.12	0.88	−0.64	0.49	0.43	0.67	0.07	0.91
Cxcl2	0.28	0.75	1.2	0.05	1.27	0.25	−1.2	0.18
Cxcl3	−2.16	0.09	0.28	0.77	0.59	0.37	2.66	0.26
Cxcl9	0.62	0.3	1.42	0	0.29	0.18	0.29	0.12
Fasl	4.5	0.14	4.94	0.07	0	1	2.42	0.11
Il12a	1.08	0.19	2.03	0	0.28	0.15	0.6	0.01
Il2	−5.96	0.42	5.65	0.42	0	1	−5.71	0.42
Il23a	−0.2	0.07	0.56	0.08	0.09	0.33	0.47	0.23
Jak3	0.83	0.14	1.59	0.01	−0.16	0.73	0.54	0.14
Lta	−0.33	0.63	3.69	0.03	1.08	0.03	−0.62	0.39
Antigen processing and presentation								
Ctss	0.48	0.12	1.07	0.01	0.51	0.1	0.51	0.06
Fcgr4	−0.23	0.58	0.97	0.06	0.78	0	−0.05	0.86
H2-DMa	−0.3	0.13	−0.3	0.3	−0.47	0.08	0.51	0.24
Ifi30	0.6	0.78	−9.1	0.18	2.11	0.28	9.59	0.35
Psmb8	4.37	0.08	5.15	0.02	0.44	0.41	1.19	0.19
Psmb9	−0.39	0.15	4.45	0.41	−0.38	0.57	0.44	0.48
Tap1	0.91	0.03	1.36	0.01	−0.11	0.74	−0.19	0.71
Tap2	2.95	0.13	4.28	0	0.62	0.21	0.96	0.11
Tapbp	2.95	0.11	1.33	0	−0.21	0.79	0.3	0.73
Inflammasome mediated signaling pathway								
Btk	2.12	0.25	2.56	0.03	2.95	0.24	1.04	0.01
Gbp2	5.16	0.12	6.02	0	−0.34	0.51	1.49	0.05
Plcg2	2.88	0.13	3.37	0.03	0.33	0.68	1.01	0.12
Trem2	2.37	0.05	2.55	0.01	2.01	0.07	1.68	0.04
Toll pathway								
Ecsit	−0.81	0.05	0.88	0.13	0.14	0.7	0.26	0.46
Fos	1.32	0.26	2.61	0.03	0.43	0.53	1.22	0.05
Ikbkb	−0.69	0	−0.21	0.14	−0.21	0	−0.14	0.12
Ikbkg	0.57	0.23	2.37	0.31	−1.93	0.29	−0.9	0.13
Map3k7	0.08	0.8	0.47	0.07	−0.02	0.85	−0.3	0.16
Mapk14	0.32	0.08	0.09	0.82	1.02	0.01	0.18	0.65
Mapk8	−1.28	0	−1.36	0.04	−0.72	0.09	−0.46	0
Nfkb1	−0.58	0.01	−0.45	0.01	−0.02	0.87	−0.07	0.13
Ppara	−2.08	0.08	−4.53	0.03	−0.71	0.38	−2.38	0.05
Cell maturation								
Ascl1	−1.64	0.21	−7.02	0.18	−0.65	0.55	−2.81	0.11
Cebpa	−0.06	0.78	0.47	0.03	0.01	0.92	0.17	0.06
Hes1	−1.6	0.01	−0.78	0.01	0.23	0.37	0.43	0.05
Id2	1.83	0.07	2.83	0	1.13	0.01	0.49	0.21
Kcnq2	−2.84	0.13	−4.58	0	0.16	0.75	−0.2	0.64
Rps6ka2	−1.64	0	−2.49	0	−0.45	0.02	−1.19	0
Runx1	1.15	0.15	1.64	0.03	0.44	0.3	1.31	0.21
Sox10	−0.64	0.04	−0.23	0.49	0.17	0.48	−0.32	0.01
Spink1	−0.11	0.91	−2.37	0.03	−0.26	0.74	0.38	0.72

^*a*^ Gene expression in ankle joint tissue at 14 and 56 days post-infection was compared to uninfected ankle joint tissue. Numbers indicate log_2_(fold change). Genes with log_2_(FC) > 0 are designated “up”; genes with log_2_(FC) < 0 are designated “down”; and genes with log_2_(FC) = 0 are designated “NC” (no change).

In contrast, C57BL/6 displayed delayed responses: complement activation peaked at 56 days (C4b: log_₂_FC = 2.11, Masp2: log_₂_FC = 0.96, and Cd55b: log_₂_FC = 0.55), and chemokine induction (Ccl24: log_₂_FC = 1.47, Ccl8: log_₂_FC = 1.15, and Lta: log_₂_FC = 1.08) occurred later. Adaptive immunity was attenuated, with Cd3d downregulation (log_₂_FC = −0.65) at 14 days and moderate Cd79a upregulation (log_₂_FC = 2.13–3.59). Both strains suppressed Il21 and Il6. These findings highlighted C3H’s hyperinflammatory arthritis susceptibility versus C57BL/6’s regulated, delayed response.

### Immune cell infiltration

**Fig 7:**
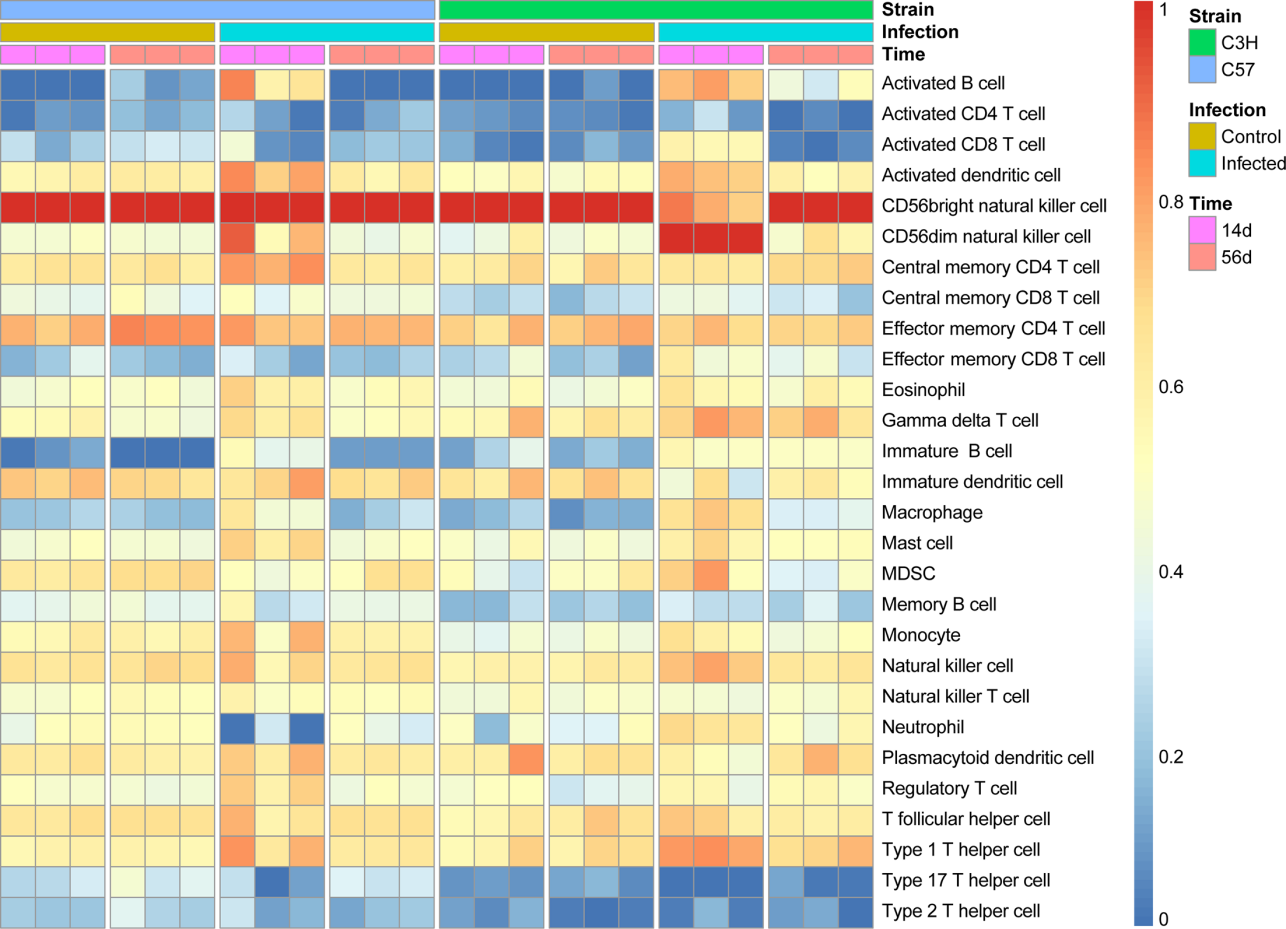
Heatmap of enrichment scores of the 28 distinct types of immune cell infiltration in the joint tissues of C57BL/6 and C3H/HeN mice on days 14 and 56 after infection.

Based on ssGSEA of transcriptomic data, 28 distinct immune cell infiltration patterns were observed in joint tissues of C57BL/6 and C3H mice following *Bb* infection, demonstrating dynamic immunological regulation (Fig. 7). Using the *t*-test method, we found that the fraction of multiple immune cells varied distinctly (Table 3). At 14 days post-infection, C3H mice had significantly elevated activated B cell (*P*.adj = 0.017), activated CD8 T cells (*P*.adj = 0.034), CD56dim natural killer cell (*P*.adj = 0.034), and macrophage (*P*.adj = 0.017), significantly reduced central memory CD4 T cell (*P*.adj = 0.021), effector memory CD4 T cell (*P*.adj = 0.022), natural killer T cell (*P*.adj = 0.017), and plasmacytoid dendritic cell (*P*.adj = 0.022), while C57BL/6 mice had significantly elevated activated B cell (*P*.adj = 0.022), immature B cell (*P*.adj = 0.028), and macrophage (*P*.adj = 0.034), and significantly reduced MDSC (*P*.adj = 0.017) and T follicular helper cell (*P*.adj = 0.022). At 56 days post-infection, C3H mice had significantly elevated immature B cell (*P*.adj = 0.028), regulatory T cell (*P*.adj = 0.043), and macrophage (*P*.adj = 0.034), and significantly reduced immature dendritic cell (*P*.adj = 0.034) and T follicular helper cell (*P*.adj = 0.021), while C57BL/6 mice had significantly elevated immature B cell (*P*.adj=0.017) and Type 1 T helper cell (*P*.adj = 0.021).

**TABLE 3:** Landscape of 28 immune cell subpopulation infiltration in LA using the ssGSEA tool^*a*^

Immune cell types	C57/B6 mice						C3H mice					
	14 days			56 days			14 days			56 days		
	Control	Infected	*P*.adj	Control	Infected	*P.*adj	Control	Infected	*P*.adj	Control	Infected	*P*.adj
Activated B cell	0	0.045 ± 0.003	0.022	0.010 ± 0.005	0	0.194	0	0.047 ± 0.002	0.017	0.003 ± 0.005	0.032 ± 0.008	0.055
Activated CD4 T cell	0.005 ± 0.003	0.008 ± 0.007	0.613	0.012 ± 0.002	0.009 ± 0.007	0.707	0.007 ± 0.003	0.011 ± 0.006	0.553	0.004 ± 0.003	0.002 ± 0.003	0.503
Activated CD8 T cell	0.016 ± 0.006	0.011 ± 0.012	0.671	0.023 ± 0.002	0.015 ± 0.001	0.055	0.006 ± 0.006	0.035 ± 0.003	0.034	0.009 ± 0.004	0.002 ± 0.002	0.236
Activated dendritic cell	0.044 ± 0.001	0.052 ± 0.003	0.141	0.045 ± 0.001	0.046 ± 0.004	0.762	0.045 ± 0.002	0.047 ± 0.002	0.533	0.044 ± 0.001	0.042 ± 0.004	0.597
CD56bright natural killer cell	0.079 ± 0.004	0.067 ± 0.009	0.294	0.077 ± 0.003	0.079 ± 0.003	0.587	0.088 ± 0.010	0.050 ± 0.004	0.061	0.089 ± 0.007	0.077 ± 0.001	0.236
CD56dim natural killer cell	0.036 ± 0.000	0.048 ± 0.007	0.238	0.034 ± 0.001	0.033 ± 0.002	0.707	0.039 ± 0.006	0.064 ± 0.005	0.034	0.039 ± 0.001	0.043 ± 0.007	0.602
Central memory CD4 T cell	0.050 ± 0.002	0.053 ± 0.006	0.587	0.048 ± 0.003	0.049 ± 0.002	0.705	0.055 ± 0.002	0.040 ± 0.002	0.021	0.055 ± 0.003	0.053 ± 0.002	0.473
Central memory CD8 T cell	0.031 ± 0.003	0.029 ± 0.003	0.602	0.032 ± 0.005	0.033 ± 0.000	0.835	0.022 ± 0.002	0.025 ± 0.000	0.212	0.020 ± 0.005	0.021 ± 0.006	0.935
Effector memory CD4 T cell	0.059 ± 0.003	0.050 ± 0.004	0.141	0.064 ± 0.001	0.059 ± 0.003	0.194	0.061 ± 0.003	0.045 ± 0.002	0.022	0.065 ± 0.003	0.053 ± 0.002	0.063
Effector memory CD8 T cell	0.019 ± 0.008	0.014 ± 0.006	0.602	0.013 ± 0.002	0.015 ± 0.003	0.494	0.026 ± 0.007	0.032 ± 0.006	0.516	0.015 ± 0.005	0.028 ± 0.006	0.144
Eosinophil	0.037 ± 0.001	0.041 ± 0.002	0.181	0.036 ± 0.002	0.040 ± 0.002	0.194	0.040 ± 0.000	0.036 ± 0.003	0.362	0.039 ± 0.003	0.040 ± 0.004	0.755
Gamma delta T cell	0.042 ± 0.000	0.043 ± 0.003	0.952	0.034 ± 0.002	0.040 ± 0.001	0.105	0.052 ± 0.005	0.048 ± 0.005	0.503	0.053 ± 0.002	0.054 ± 0.005	0.945
Immature B cell	0.005 ± 0.004	0.028 ± 0.002	0.028	0	0.007 ± 0.000	0.017	0.020 ± 0.010	0.032 ± 0.003	0.334	0.014 ± 0.003	0.037 ± 0.001	0.028
Immature dendritic cell	0.056 ± 0.002	0.047 ± 0.010	0.465	0.051 ± 0.002	0.053 ± 0.002	0.493	0.057 ± 0.001	0.029 ± 0.010	0.139	0.059 ± 0.003	0.044 ± 0.003	0.034
MDSC	0.048 ± 0.002	0.032 ± 0.002	0.017	0.052 ± 0.003	0.048 ± 0.006	0.533	0.035 ± 0.012	0.043 ± 0.007	0.569	0.047 ± 0.007	0.029 ± 0.007	0.12
Macrophage	0.016 ± 0.001	0.033 ± 0.003	0.034	0.014 ± 0.002	0.017 ± 0.006	0.602	0.015 ± 0.004	0.043 ± 0.003	0.017	0.010 ± 0.003	0.026 ± 0.002	0.034
Mast cell	0.036 ± 0.001	0.044 ± 0.003	0.141	0.033 ± 0.001	0.036 ± 0.002	0.181	0.041 ± 0.003	0.039 ± 0.003	0.553	0.039 ± 0.002	0.039 ± 0.001	0.785
Memory B cell	0.030 ± 0.001	0.024 ± 0.007	0.465	0.030 ± 0.002	0.031 ± 0.001	0.587	0.017 ± 0.004	0.018 ± 0.002	0.707	0.018 ± 0.003	0.019 ± 0.006	0.86
Monocyte	0.044 ± 0.001	0.043 ± 0.007	0.907	0.044 ± 0.002	0.045 ± 0.002	0.613	0.035 ± 0.001	0.037 ± 0.003	0.475	0.038 ± 0.001	0.035 ± 0.003	0.475
Natural killer T cell	0.037 ± 0.001	0.035 ± 0.002	0.246	0.040 ± 0.001	0.040 ± 0.002	0.785	0.040 ± 0.002	0.028 ± 0.001	0.017	0.041 ± 0.000	0.038 ± 0.004	0.519
Natural killer cell	0.050 ± 0.002	0.044 ± 0.003	0.142	0.051 ± 0.004	0.050 ± 0.000	0.845	0.050 ± 0.006	0.047 ± 0.002	0.705	0.053 ± 0.001	0.048 ± 0.002	0.146
Neutrophil	0.037 ± 0.005	0.008 ± 0.014	0.156	0.040 ± 0.001	0.032 ± 0.008	0.462	0.032 ± 0.015	0.041 ± 0.003	0.533	0.035 ± 0.009	0.037 ± 0.006	0.785
Plasmacytoid dendritic cell	0.050 ± 0.001	0.046 ± 0.005	0.503	0.045 ± 0.000	0.048 ± 0.002	0.246	0.057 ± 0.005	0.033 ± 0.003	0.022	0.056 ± 0.001	0.052 ± 0.004	0.473
Regulatory T cell	0.037 ± 0.004	0.044 ± 0.003	0.212	0.033 ± 0.001	0.036 ± 0.004	0.51	0.042 ± 0.004	0.031 ± 0.004	0.12	0.030 ± 0.002	0.040 ± 0.003	0.043
T follicular helper cell	0.050 ± 0.001	0.043 ± 0.001	0.022	0.050 ± 0.002	0.052 ± 0.002	0.533	0.049 ± 0.002	0.042 ± 0.002	0.084	0.057 ± 0.001	0.045 ± 0.002	0.021
Type 1T helper cell	0.044 ± 0.002	0.049 ± 0.003	0.207	0.043 ± 0.001	0.048 ± 0.001	0.021	0.052 ± 0.003	0.051 ± 0.002	0.947	0.056 ± 0.002	0.054 ± 0.003	0.533
Type 17T helper cell	0.022 ± 0.002	0.008 ± 0.008	0.227	0.028 ± 0.005	0.025 ± 0.002	0.569	0.007 ± 0.002	0	0.091	0.010 ± 0.004	0.004 ± 0.004	0.305
Type 2T helper cell	0.016 ± 0.002	0.012 ± 0.005	0.473	0.021 ± 0.005	0.013 ± 0.003	0.246	0.009 ± 0.003	0.004 ± 0.005	0.465	0.002 ± 0.001	0.006 ± 0.005	0.475

^*a*^ Numbers indicate the calculated ssGSEA enrichment score of immune cell signature genes in immune cells; *P*.adj: the adjusted *P*-value.

### Immunohistochemistry

**Fig 8:**
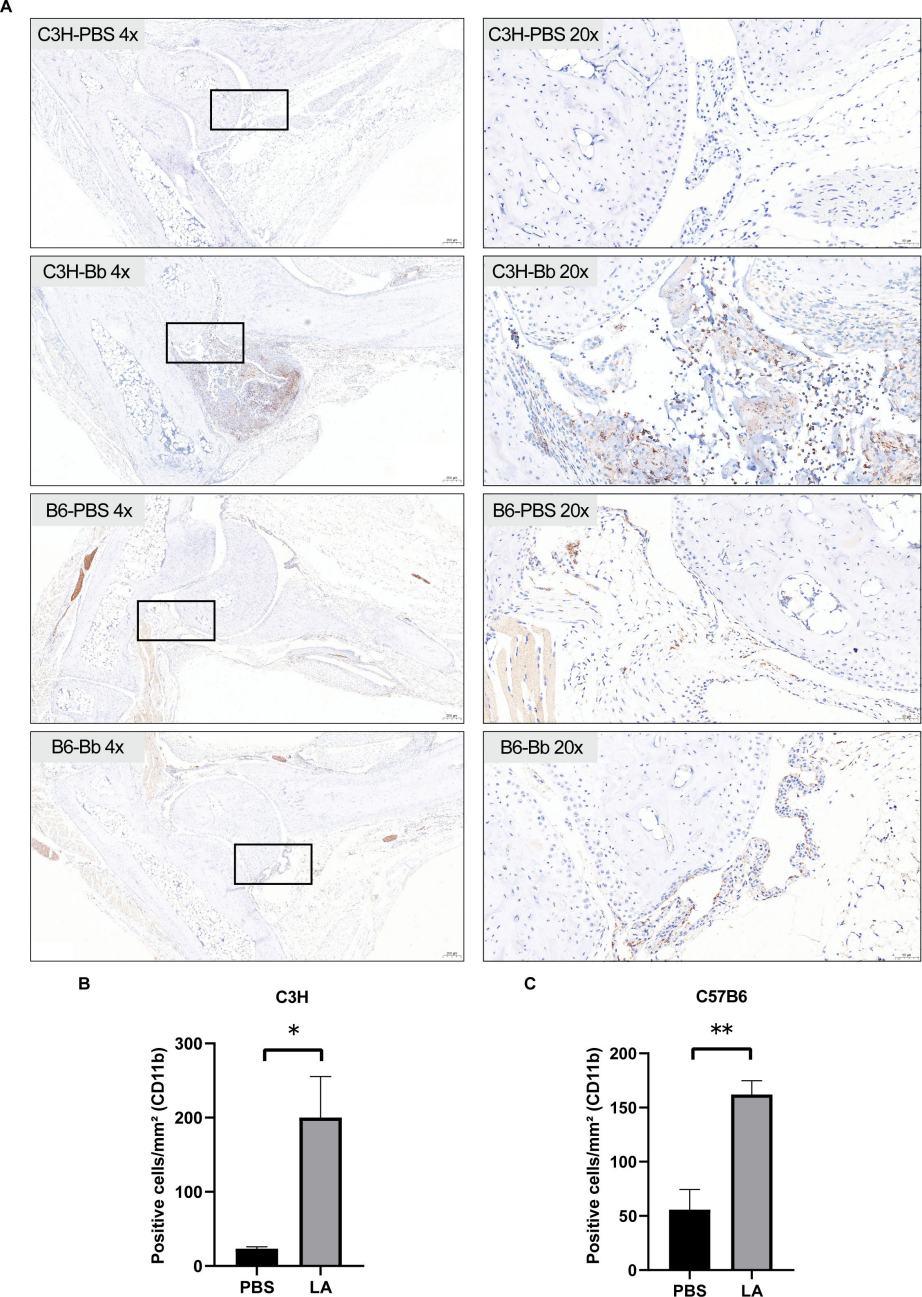
Representative immunohistochemical staining of CD11b in articular tissues. (**A**) CD11b immunoreactivity within synovial tissue of C3H and C57BL/6 murine models on day 14 after infection; the left panels are at 4× magnification, while the right panels are magnified at 20×, showcasing detailed enlargements of specific regions within the 4× images. Regarding the rectangular areas detected by the forensic tools, this is attributable to our utilization of whole slide imaging in pathology, which constructs digital imaging by stitching together square fields of view and supports free magnification from 0× to 400×. Blank areas within the scanning range (where there is no tissue on the slide) are identified as a white background. (**B and C**) Statistical assessment of CD11b-positive cell density in experimental and control cohorts of C3H and C57BL/6 murine models on day 14 after infection, respectively. Data are presented as mean ± SEM and quantified (*n* = 3, error bars represent the SEM, mean ± SEM, *t*-test, compared with the control group, *P < 0.05 and **P < 0.01).

CD11b immunoreactivity in the synovial tissue of C3H and C57BL/6 mice was evaluated by immunohistochemistry on day 14 post-infection (Fig. 8). In C3H mice, the CD11b-positive cell density was significantly higher in the infected group than in the control group (23 ± 2.517 versus 200 ± 55.43, *P*-value < 0.05). C57BL/6 mice also showed significantly elevated CD11b-positive cell density between the infected group and the control group (55.67 ± 18.77 versus 162 ± 12.77, *P*-value < 0.05).

## DISCUSSION

Lyme arthritis, caused by *Borrelia burgdorferi*, exhibits host-genetic-dependent severity. To investigate molecular mechanisms underlying these differences, we compared two mouse strains with distinct disease severities: C3H (severe) and C57BL/6 (B6, mild). Phenotypic analysis revealed a clear hierarchy: C3H mice developed significant joint swelling and mobility limitations, consistent with their known sensitivity to experimental LA (29), while C57BL/6 mice showed mild symptoms. X-ray and histopathology confirmed this gradient, particularly in soft tissue edema and inflammatory cell infiltration. Transcriptomic analysis identified divergent host-response patterns: C3H mice displayed acute-phase cytokine storms transitioning to chronic dysregulation, whereas C57BL/6 mice exhibited acute robust suppression followed by gradual restoration. These patterns highlight immune-response diversity and provide insights into LA host-pathogen dynamics.

Enrichment analysis (GO/KEGG) revealed shared and strain-specific immune regulation. Both strains showed sustained antigen presentation activation (Golgi/MHC pathways) and continuous T-cell responses (β2-microglobulin/CD8 binding). Common enrichment of autoimmune disease pathways (viral myocarditis, thyroiditis, and type I diabetes) suggested potential immune cross-reactivity or persistent inflammation (30–32). Strain-specific variations included C3H-selective MHC Ib hyperactivation driving cytotoxic lymphocyte expansion (33), and C57BL/6-unique dynamic peptide binding with TRAIL-mediated apoptosis enabling phase-specific containment (34, 35).

Our ssGSEA analysis revealed distinct immune trajectories. In C3H mice, acute cytotoxic hyperactivation (CD8^+^T, NK cells) transitioned to chronic macrophage persistence and Treg dominance, forming a self-sustaining inflammatory niche. CD8^+^T depletion markedly reduced LA severity (36). Elevated joint macrophages on days 14 and 56 (37) implied that macrophage plasticity is essential for resolution, warranting future flow cytometry analysis of M1/M2 dynamics. Increased Tregs on day 56 correlated with reduced joint edema. Treg depletion pre-infection exacerbated swelling and histopathology with diminished IFN-γ/IL-10 (38), confirming Tregs’ critical containment role. C3H-specific dendritic cell dysregulation—reduced acute-phase pDCs and chronically depleted immature DCs—indicated compromised antigen processing (39), driving persistent bacterial colonization and progressive inflammation (40, 41). Conversely, C57BL/6 mice achieved acute control via Tfh suppression and MDSC reduction, transitioning to chronic Th1-mediated repair. Confirmed Tfh dysfunction in C57BL/6 mice disrupted germinal centers, complement deposition, and antibody maturation—key reinfection susceptibility factors (42). While MDSCs remain poorly characterized in LA (43), Th1 cells demonstrated protective efficacy by resolving murine carditis through cell-mediated immunity (44). These phenotypic divergences likely stem from differential genetics, cytokine networks, transcriptional regulation, and immune evasion tactics (10, 45–48). Neutrophils appear to contribute to LA pathogenesis through cytokine production (including TNF-α, MCP-1, and IL-10), which may facilitate endothelial activation and potentially assist *Bb* dissemination via transcellular routes (49). Current evidence suggests that neutrophil depletion could intensify pathology by disrupting immune equilibrium (50). Prior research indicated that *Bb* infection primarily mobilizes neutrophils through cellular accumulation mechanisms rather than transcriptional activation—a phenomenon consistent with their terminally differentiated biology (23). Our integrated ssGSEA and IHC analyses further suggested that transcriptional profiling may underestimate neutrophil infiltration in LA, likely reflecting their RNA-deficient nature (51, 52), whereas IHC provided more definitive evidence of recruitment. Future investigations using flow cytometry could help elucidate the spatiotemporal dynamics of neutrophil involvement more comprehensively.

Gene set analysis revealed distinct immune response profiles across mouse strains after *Borrelia burgdorferi* infection. We focused on changes in adaptive immunity gene sets, complement activation, chemokine/cytokine pathways, and innate immune networks. Compared to C57BL/6 mice, C3H mice showed significantly greater upregulation of adaptive immune genes by day 14 post-infection, including coordinated induction of T cell genes (Cd3d, Cd3g, and Cd4), B cell markers (Cd79a), costimulatory molecules (Cd28 and Cd40), and checkpoint inhibitor Ctla4. Previous studies indicate that impaired early adaptive immunity may trigger prolonged inflammation, potentially leading to chronic LA despite antibiotics (53, 54). C3H mice’s robust adaptive response suggests effective pathogen recognition and immune mobilization. Complement activation differed between strains: C3H mice exhibited rapid, peak activation by day 14, while C57BL/6 mice showed gradual activation lasting 56 days, possibly explaining early joint pathology differences. C3-deficient C57BL/6 mice maintained high spirochete loads in joint and ear tissues (55), confirming complement’s essential role in controlling infection. Lyme spirochetes evade complement through multiple mechanisms, including surface lipoproteins (e.g., ElpB/ElpQ) and C4bp-P43 receptor exploitation, enabling chronic infection (56–58). These findings highlight complement’s critical role in governing initial pathogen spread and disease progression in Lyme borreliosis.

Chemokines and cytokines critically influence LA pathogenesis by orchestrating immune cell recruitment and activation (59, 60). Our study identified strain-dependent variations: in C3H mice, neutrophil-associated chemokines (Cxcl1/Cxcl2) surged by day 14 (61), corresponding to their severe acute joint swelling and increased neutrophil infiltration shown by ssGSEA. Subsequent upregulation of Ccl5, Ccl8, and Csf2 suggests that these chemokines coordinate monocyte/macrophage accumulation, amplifying pathology (62–65). Conversely, C57BL/6 mice suppressed cytokine signaling (notably Ccl24/Csf3), indicating effective immunoregulation (66, 67). Innate immunity differences further explain arthritis susceptibility: C3H mice displayed early hyperactivation (day 14) via excessive TLR12/TLR2 signaling (36, 68, 69) and elevated inflammasomes (Mefv/Nlrp3) (35, 70, 71), where compensatory anti-inflammatory signals (PD-L1/IL-10/Tgfb1) failed to resolve inflammation (72–74). C57BL/6 mice exhibited delayed TLR2 activation with early upregulation of the inhibitor Irak3 (75, 76), revealing a potential protective mechanism balancing inflammation and tissue repair.

However, several directions remain for further exploration: integrating multi-omics approaches (transcriptomics, proteomics, and metabolomics) to analyze host-pathogen dynamics; expanding sample sizes to improve reliability; quantifying pathogen loads at infection sites to link bacterial burden with gene expression and immune response intensity; and confirming strain-specific immune characteristics in human populations for personalized treatments. A key limitation is persistent bone marrow contamination affecting joint tissue analyses, which future studies will address through flow cytometry and single-cell sequencing to better distinguish articular and hematopoietic cells. These investigations will advance understanding of Lyme disease pathogenesis and therapeutic development.

### Conclusion

Through comparative transcriptome analysis, this study revealed significant strain-dependent immune response characteristics in two mouse strains during early (14 days) and late (56 days) stages of *Borrelia burgdorferi* infection. Specifically, C3H mice maintained persistent inflammatory activation, including sustained inflammasome activation and chemokine signaling expression, consistent with their severe arthritis phenotype; C57BL/6 mice demonstrated resistance to severe pathology by maintaining moderate immune activation and effective homeostatic regulation. Notably, distinct strain-specific regulatory patterns were observed in key immune pathways, including inflammasome components (especially NLRP3), chemokine networks (CCR/CXCR families), and adaptive immune responses. These findings not only elucidate the mechanisms by which host genetic background influences LA susceptibility but also suggest that treatment strategies should consider individual genetic differences.

## Data Availability

Raw data have been deposited to the National Center for Biotechnology Information (NCBI) under BioProject accession number PRJNA1414168.
